# Functional fitness norms and trends of community-dwelling older adults in urban China

**DOI:** 10.1038/s41598-021-97320-5

**Published:** 2021-09-07

**Authors:** Yanan Zhao, Zhuying Wang, Pak-Kwong Chung, Sheng Wang

**Affiliations:** 1 School of Sports Science and Physical Education, Nanjing Normal University, Nanjing, 210023 China; 2 Department of Sports, Physical Education and Health, Hong Kong Baptist University, Hong Kong, China; 3 Department of Rehabilitation Medicine, The Affiliated Suzhou Science and Technology Town Hospital of Nanjing Medical University, Nanjing Medical University, Suzhou, 215153 China

**Keywords:** Health care, Risk factors, Signs and symptoms

## Abstract

This study aimed to (1) establish age- and sex-specific functional fitness (FF) norms in Chinese older adults living in urban communities and (2) explore ageing-related degradations and sex differences in each FF dimension. A pool of 2398 individuals (1128 men; 1270 women) aged 60–98 years were recruited from urban communities of Nanjing, China. FF was measured using the Senior Fitness Test battery. FF norms were established for men and women in 5-year age intervals using five percentiles (10th, 25th, 50th, 75th, 90th). Significant degradations in FF were correlated with increases in age. Around half of test items in 75–79 years group (3 for men; 4 for women) and over half in 80–84 years group (5 for men; 4 for women) exhibited significant decreases in FF compared with the adjacent younger group, indicating that ages of 75 and 80 years are two potential turning points in FF declines. Significant differences existed between the overall FF of men and women; women outperformed men in flexibility and men outperformed women in upper body strength, agility/dynamic balance, and aerobic endurance. Significant age and sex effects occurred in each fitness dimension, which helps individualised program design and promotes an active lifestyle in older adults.

## Introduction

Accompanied by a decline in fertility and increases in longevity, population ageing is occurring worldwide^1^. China as a developing country, has the largest population on Earth. Nevertheless, China has the fastest rate of population ageing. The percentage of people aged ≥ 60 years in China has increased from 8.5 to 17.4% in the past 30 years, and the percentage is expected to reach 24.8% by 2050^2^. Such a demographic shift has brought and will continue to bring various challenges, and understanding how to maintain and improve the wellbeing of older adults is a pertinent concern^3^.

Because of the inevitable decline in fitness that occur as a person ages, every person faces risks of functional decline and frailty, which inevitably result in the loss of independence in activities of daily living^4^. Multiple studies have highlighted the importance of living independently to older adults’ wellbeing, especially in their later years^5^. A certain level of physical fitness is necessary for maintaining physical independence and preventing disability^6^. Some studies have indicated that functional fitness (FF), which is defined as the physiological capacity to perform normal everyday activities safely, independently, and without undue fatigue^7^, improves and maintains older adults’ wellbeing and quality of life^8,9^. Therefore, researchers should strive to gain a comprehensive understanding of the FF levels of older adults.

To date, many countries and regions have established FF norms for older adults^10–13^. Due in part to the genetic and ethnical heterogeneity, cross-cultural comparisons in normative FF values have revealed significant differences in the fitness performance of older adults of the same age^12^. Our previous study indicated that Hong Kong’s Chinese older adults performed worse than American older adults did in all fitness components except for muscle strength of the lower extremities^10^. However, Hong Kong is one of China’s special administrative regions, and distinct cultural and social discrepancies exist between mainland China and Hong Kong. The FF level of older adults in mainland China and what the pattern of fitness degradation with age remained unknown. Although there is yearly National Physical Fitness Test for older adults in mainland China, the applicable age range is 60 to 69 years, which limits the ability to obtain knowledge and an understanding of the fitness of a broader age group.

The present study therefore aimed to establish the FF norms of Chinese adults aged ≥ 60 years who reside in urban communities and to identify the degradation pattern of each FF dimension with increases of age. The results from this study can elucidate the current FF situation of Chinese older adults, which may help policymakers draft health-related regulations and assist clinical practitioners with initialising exercise programmes for the early prevention of frailty. Moreover, older adults can use the FF normative scores for interpersonal and intrapersonal comparisons that could inspire them to lead a more active lifestyle.

## Methods

### Study design

This was a population-based cross-sectional study conducted in Nanjing, China. Nanjing is a well-known historic city located in eastern China. It is the capital of Jiangsu province and one of China’s 15 sub-provincial cities. Unlike the most commonly studied super cities such as Shanghai, Beijing and Guangzhou, Nanjing is a well-representative city of China with the typical characteristics associated with rapidly urbanising areas^14^. By the end of 2019, the permanent population of Nanjing was over 7 million, and 22% of individuals were aged ≥ 60 years^15^.

### Participants

A total of 3,326 community-dwellers aged ≥ 60 years were reached through flyers and activities in urban community senior service centres. People who could not walk independently or had any severe diseases (e.g., congestive heart failure, uncontrolled high blood pressure, or dizziness) that would limit their participation in physical tests were excluded. All participants gave written informed consent in accordance with the Declaration of Helsinki. This study was approved by the Committee on the Use of Human and Animal Participants in Teaching and Research of Nanjing Normal University.

### Measurements

FF was measured using the Senior Fitness Test (SFT) battery, which was developed and validated by Rikli and Jones for the early identification of older adults at risk of losing functionality^13^. The SFT battery is feasible and widely used for evaluating FF of older population^16^. It contains seven test items measuring five fitness dimensions that are highly associated with the functional mobility of independent older adults. The seven test items include the 30-s chair stand (CS) test for lower body strength, the 30-s arm curl (AC) test for upper body strength, the back scratch (BS) test for upper body flexibility, the chair sit-and-reach (SR) test for lower body flexibility, the 8-ft up-and-go (UG) test for motor agility and dynamic balance, the 2-min step-in-place (Step) test for aerobic endurance, and the body mass index (BMI) assessment for body composition. All the tests were conducted with strict adherence to the instructions of the SFT Manual^17^.

### Test procedure

Participants who signed the consent form were asked to wear suitable clothes and shoes to complete the tests during a scheduled time slot. More than 90% of the tests were conducted between 9:00 am and 11:30 am and between 2:00 pm and 4:30 pm, and each group had up to 30 participants. Each participant had a 5 to 10-min rest before their resting blood pressure (BP) and heart rate were recorded onsite. With the consideration of safety, participants having systolic BP over 160 mmHg were not allowed to attend the test. Participants with the systolic BP ≤ 160 mmHg were instructed to perform a 3 to 5 min warm-up exercise routine that included walking around and stretching. They then began the test while following a generally nonspecific test order; the only sequence requirement was that the Step test be administered after a participant had completed all tests. Out of concern for data precision and participant safety, each test station had at least one tester who assessed fitness performance and one student helper who assisted the tester in test administration. A total of 23 testers with exercise science education backgrounds were recruited to collect data. To ensure protocol consistency and minimise testing errors, testers completed a standardised 2-h training workshop conducted by the leader of this project. All tests were conducted in local senior service centres in the participants’ communities. Data were collected from October 2018 to September 2019.

### Data analysis

All statistical analyses were conducted using SPSS v24.0 (IBM, Armonk, NY, USA), and the significance level was less than 0.05. Data were entered and double-checked by different research assistants to ensure the accuracy of data inputs. Considering that some participants had extremely different abilities in the test and they would have represented some groups in reality, only data that lay outside the bounds of ‘third quartile + 3 × interquartile range’ or ‘first quartile − 3 × interquartile range’ were set aside.

Participants were divided into 5 age groups (AGs) (i.e., AG1 = 60–64 years, AG2 = 65–69 years, AG3 = 70–74 years, AG4 = 75–79 years, AG5 = 80–84 years, AG6 = 85 years and over) according to the participants’ sex. Five percentiles (i.e., 10th, 25th, 50th, 75th, and 90th) were applied in the construction of the percentile grid.

A two-way analysis of variance (ANOVA) was conducted to explore the interactions of AGs and sex in each fitness test. Sex differences in overall FF for each age group were examined using a one-way multivariate analysis of variance (MANOVA). Researchers also performed a one-way ANOVA to examine age effect in each fitness component for women and men, respectively. A planned contrast was then performed to reveal the differences between any two adjacent AGs. The level of statistical significance was therefore adjusted to < 0.01 given that 5 comparisons were made in the analysis. Effect size (ES) was calculated using Hedges’ *g*, where *g* is the mean difference of 2 comparison groups divided by the weighted and pooled standard deviation^18^. An ES of ≤ 0.20 was considered low, an ES of ≥ 0.80 was considered high, and an ES between 0.20 and 0.80 was considered moderate^19^. The overall declining trend for each fitness dimension was calculated according to the equation of (AG_6_ − AG_1_)/AG_1_ × 100%, except for the flexibility test where the equation is (AG_6_ − AG_1_)/AG_6_ × 100%.

## Results

### Participant flow and characteristics

A total of 2567 older adults were qualified and enrolled in this study, and the data of 169 participants were set aside because they showed missing values in more than 4 out of 7 tests. After extreme outliers were deleted, the data of 2398 participants (1128 men and 1270 women) remained for data analysis. The demographic characteristics of participants are detailed in Table 1.

**Table 1:** Demographic characteristics of participants (*n* = 2398).

	Combined	60–64	65–69	70–74	75–79	80–84	≥ 85
**Number**							
Men	1128	229	246	226	219	165	42
Women	1270	276	303	245	221	164	61
**Age (years)**							
Men	71.8 (7.50)	61.9 (1.42)	66.9 (1.47)	72.0 (1.45)	76.9 (1.41)	81.8 (1.41)	87.4 (2.90)
Women	71.5 (7.49)	62.1 (1.45)	67.1 (1.52)	71.9 (1.48)	76.8 (1.39)	81.8 (1.44)	86.8 (1.75)
**Weight (kg)**							
Men	68.1 (10.2)	70.9 (9.50)	68.8 (9.94)	69.2 (9.91)	67.0 (10.9)	63.7 (9.00)	65.9 (10.5)
Women	57.7 (9.49)	60.2 (8.84)	59.1 (9.15)	58.4 (9.41)	56.1 (9.11)	54.0 (9.85)	52.2 (8.73)
**Height (cm)**							
Men	165.4 (6.69)	168.0 (6.50)	166.0 (6.10)^#^	165.6 (6.62)	164.1 (6.51)	162.6 (6.75)	164.1 (6.43)
Women	153.0 (6.11)	156.2 (5.53)	154.0 (5.64)	153.2 (5.80)	151.3 (5.54)^#^	150.0 (5.42)^#^	147.6 (5.74)
**Systolic blood pressure (mmHg)**							
Men	138.9 (18.8)	136.5 (18.9)	138.1 (16.6)	140.0 (18.4)	139.2 (20.9)	140.4 (18.9)	146.0 (19.3)
Women	137.1 (16.8)	132.0 (15.8)	134.7 (16.1)	138.8 (16.4)*	141.9 (16.5)*	141.2 (17.1)*	135.9 (17.8)
**Diastolic blood pressure (mmHg)**							
Men	78.7 (11.7)	85.1 (10.9)	80.1 (10.8)^#^	77.7 (11.3)	75.3 (10.9)	73.5 (10.6)	78.8 (13.7)
Women	73.9 (9.90)	76.2 (9.09)	74.7 (9.20)	73.8 (9.27)	72.5 (10.4)	71.9 (11.1)*	69.5 (11.0)
**Resting heart rate (b/m)**							
Men	75.0 (12.1)	77.4 (12.4)	75.5 (11.9)	74.3 (12.4)	74.0 (10.9)	73.8 (12.7)	74.5 (11.5)
Women	74.2 (10.8)	74.2 (8.87)	74.6 (10.4)*	73.2 (11.2)*	74.1 (12.2)*	74.1 (11.8)*	76.5 (9.95)*

Dara are presented as mean (SD).

*Comparing with men at *p* < 0.05.

^#^Comparing with adjacent younger group at *p* < 0.05.

### Sex and age effects on FF components

Table 2 displays descriptive analysis of each FF component. Results from the two-way ANOVA revealed significant interaction effects of sex and AG in the following tests: BS, *F*(5, 2333) = 2.31, *p* = 0.042; UG, *F*(5, 2343) = 4.09, *p* = 0.001; and Step, *F*(5, 2195) = 5.94, *p* < 0.001. No significant interaction effects were found in the AC, CS, or SR, nor were such effects found in participants’ BMIs.

**Table 2:** Age-group based means and differences of each functional fitness test for men and women.

	Age groups						Group differences					
	60–64	65–69	70–74	75–79	80–84	≥ 85	*ES*^a^	*ES*^b^	*ES*^c^	*ES*^d^	*ES*^e^	%Decline^f^
**30 s Arm curl test (reps.)**												
Men	22.7 (4.57)	18.3 (5.86)	17.0 (6.32)	15.6 (5.56)	13.4 (4.83)	13.1 (4.45)	0.833*	0.214*	0.235*	0.419*	0.063	42.3
Women	20.5 (4.27)	16.9 (5.58)	15.8 (4.91)	14.8 (5.04)	13.4 (5.30)	13.1 (4.56)	0.720*	0.208*	0.201	0.272*	0.059	36.1
ES	0.499*	0.245*	0.213*	0.151	0	0						
**30 s Chair stand test (reps.)**												
Men	16.9 (3.84)	16.9 (4.68)	15.3 (4.11)	14.6 (4.08)	12.8 (3.50)	11.6 (3.96)	0	0.362*	0.171	0.469*	0.335	31.4
Women	16.9 (3.42)	16.2 (4.34)	15.7 (4.29)	14.0 (4.23)	12.8 (3.91)	11.7 (3.54)	0.178	0.116	0.399*	0.293*	0.288	30.1
ES	0	0.156*	0.095	0.144	0	0.008						
**Back scratch test (cm)**												
Men	− 11.5 (12.0)	− 10.0 (13.2)	− 12.8 (12.9)	− 14.8 (13.4)	− 14.6 (14.5)	− 23.3 (12.7)	0.119	0.214	0.152	0.014	0.614*	102.6
Women	− 3.74 (10.5)	− 6.46 (11.3)	− 7.84 (12.0)	− 9.01 (12.5)	− 10.2 (12.6)	− 12.9 (12.6)	0.250*	0.119	0.096	0.096	0.214	244.9
ES	0.693*	0.291*	0.399*	0.447*	0.323*	0.427*						
**Chair sit and reach test (cm)**												
Men	− .300 (10.3)	− 1.17 (11.1)	− 1.89 (12.4)	− 3.79 (11.5)	− 8.08 (12.2)	− 11.6 (13.2)	0.081	0.064	0.159	0.363*	0.284	2593
Women	5.90 (9.31)	6.01 (9.36)	4.40 (10.7)	1.76 (11.1)	.004 (9.78)	− 2.72 (9.52)	0.012	0.161	0.242*	0.166	0.280	146.1
ES	0.634*	0.706*	0.545*	0.491*	0.729*	0.393*						
**8ft Up and go test (s)**												
Men	5.56 (1.18)	5.60 (1.61)	6.18 (1.97)	6.71 (1.97)	7.69 (2.16)	8.94 (2.56)	0.028	0.324*	0.269*	0.477*	0.558*	37.8
Women	5.22 (1.19)	5.91 (1.49)	6.43 (1.85)	7.34 (2.20)	8.24 (2.37)	9.62 (3.14)	0.511*	0.313*	0.450*	0.396*	0.532*	45.7
ES	0.287*	0.201*	0.131	0.302*	0.243	0.058						
**2 min Step test (reps.)**												
Men	96.7 (17.5)	96.1 (20.0)	93.1 (18.4)	88.1 (20.5)	77.1 (21.7)	77.5 (19.1)	0.032	0.156	0.257*	0.523*	0.019	19.9
Women	103.7 (18.4)	93.6 (20.2)	92.1 (20.0)	84.6 (20.4)	77.5 (21.4)	66.6 (18.9)	0.521*	0.075	0.372*	0.341*	0.523*	35.8
ES	0.389*	0.124	0.052	0.171	0.019	0.409*						
**Body mass index (kg/m**^**2**^**)**												
Men	25.1 (2.74)	24.9 (3.06)	25.2 (3.16)	24.9 (3.59)	24.1 (2.92)	24.2 (3.39)	0.069	0.097	0.089	0.241	0.033	3.59
Women	24.6 (3.19)	24.9 (3.53)	24.9 (3.64)	24.5 (3.65)	24.1 (3.95)	24.0 (4.19)	0.089	0	0.110	0.106	0.025	2.44
ES	0.167	0	0.088	0.110	0	0.014						

*ES* effect size.

*p < 0.05.

^a^60–64 age group versus 65–69 age group.

^b^65–69 age group versus 70–74 age group.

^c^70–74 age group versus 75–79 age group.

^d^75–79 age group versus 80–84 age group.

^e^80–84 age group versus ≥ 85 age group.

^f^The percentage of declining when comprising ≥ 85 age group with 60–64 age group.

A one-way MANOVA was conducted to examine sex effects in overall FF, and the results indicated significant sex differences in the overall FF of each age group (all *p* < 0.001). Additionally, women outperformed men in the BS (ES = 0.291–0.693) and SR (ES = 0.393–0.729) in each age group (all *p* < 0.05). Men outperformed women in the AC tests in AG1, AG2, and AG3 (ES = 0.213–0.499); in the UG test in AG1, AG2, and AG4 (ES = 0.201–302); and in the Step test in AG1 (ES = 0.389) and AG6 (ES = 0.409). Little differences were found between men and women’s performance in the CS test or between men and women’s BMIs.

Both men and women’s performances exhibited significant degradation with advanced age in each FF component. The most obvious decrease was evident in the BS and SR tests, and almost no changes in BMI values were presented. As for other fitness parameters, the UG and AC tests revealed a faster rate of decrease among men than was identified in the CS and Step tests (i.e., SR > BS > UG > AC > CS > Step); this applied to women as well (i.e., BS > SR > UG > AC > Step > CS). Further investigation into the adjacent AGs’ differences in each FF component revealed that around half of FF test items in AG4 (men = 3, women = 4) and more than half in AG5 (men = 5, women = 4) exhibited significant decreases in FF compared with the adjacent younger group, indicating that the ages of 75 and 80 years are two potential markers for ageing-associated degradation in FF.

### Sex-and age-specific FF normative scores

**Figure 1:**
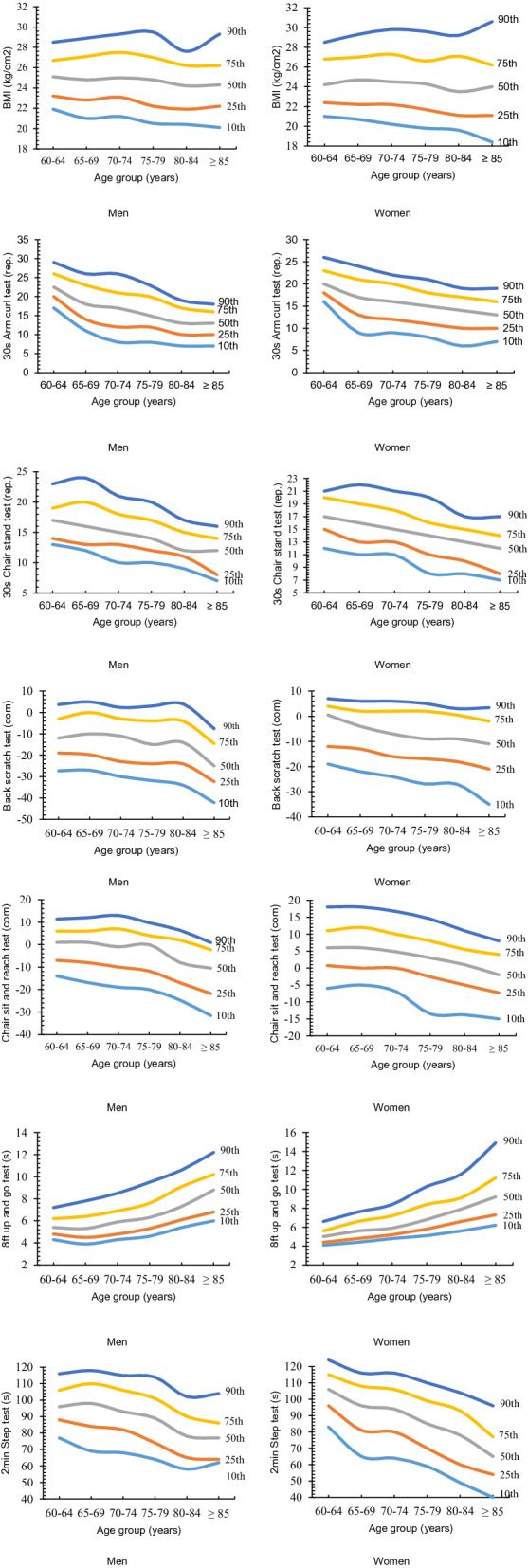
Percentile-based changing trends with age for each fitness component in men and women, respectively.

Table 3 presents the normative FF values determined using 5 percentile categories, and Fig. 1 displays the FF change trends with increases in age for men and women, respectively.

**Table 3:** Age-group percentile score of functional fitness for men and women.

	Men (n = 1128)						Women (n = 1270)					
	n	10th	25th	50th	75th	90th	n	10th	25th	50th	75th	90th
**30 s Arm curl test (reps.)**												
60–64	220	17	20	22.5	26	29	269	16	18	20	23	26
65–69	236	11	14	18	23	26	296	9	13	17	21	24
70–74	217	8	12	17	21	26	235	9	12	16	20	22
75–79	209	8	12	15	20	23	201	8	11	15	18	21
80–84	164	7	10	13	17	19	149	6	10	14	17	19
≥ 85	39	7	10	13	16	18	52	7	10	13	16	19
**30 s Chair stand test (reps.)**												
60–64	224	13	14	17	19	23	262	12	15	17	20	21
65–69	233	12	13	16	20	24	288	11	13	16	19	22
70–74	210	10	13	15	18	21	240	11	13	15	18	21
75–79	209	10	12	14	17	20	202	8	11	14	16	20
80–84	162	9	11	12	15	17	147	8	10	13	15	17
≥ 85	36	7	8	12	14	16	55	7	8	12	14	17
**Back scratch test (cm)**												
60–64	226	− 27.3	− 19.0	− 12.0	− 3.0	3.7	275	− 19.0	− 12.0	0.50	4.0	7.0
65–69	239	− 27.0	− 19.7	− 10	0	5.0	299	− 22.0	− 13.0	− 4.0	2.0	6.0
70–74	219	− 30.0	− 23.0	− 11.0	− 3.0	2.3	243	− 24.0	− 16.0	− 7.0	2.0	6.0
75–79	211	− 31.9	− 24.0	− 15.0	− 4.0	3.0	212	− 26.9	− 16.9	− 9.0	2.0	5.1
80–84	161	− 34.0	− 24.0	− 14.0	− 4.0	4	153	− 27.0	− 18.0	− 9.0	0.50	3.0
≥ 85	40	− 42.2	− 32.4	− 25.0	− 14.6	− 7.6	55	− 35.0	− 21.0	− 11.0	− 2.0	3.4
**Chair sit and reach test (cm)**												
60–64	225	− 14.0	− 7.0	1.0	6.0	11.4	270	− 6.0	0.73	6.0	11.0	18.0
65–69	240	− 16.9	− 8.0	1.0	6.0	12	299	− 5.0	0	6.0	12.0	18.0
70–74	220	− 19.0	− 10.0	− 1.0	7.0	13	242	− 6.9	0	4.8	10	16.7
75–79	216	− 20.0	− 11.8	0	4.0	9.7	214	− 13.5	− 2.6	3.0	8.0	14.5
80–84	162	− 24.7	− 17.0	− 8.0	2.0	6.4	155	− 13.8	− 5.0	1.0	5.5	11.0
≥ 85	40	− 31.6	− 21.8	− 10.5	− 2.3	0.90	54	− 15	− 7.3	− 2.0	4.0	8.0
**8ft Up and go test (s)**												
60–64	227	4.3	4.8	5.4	6.2	7.2	272	4.1	4.4	5.0	5.6	6.6
65–69	242	3.9	4.5	5.3	6.4	7.8	301	4.4	4.8	5.6	6.6	7.6
70–74	219	4.3	4.8	5.9	6.9	8.5	242	4.8	5.2	5.9	7.2	8.4
75–79	215	4.6	5.3	6.3	7.6	9.5	214	5.1	5.8	6.8	8.4	10.3
80–84	162	5.4	6.1	7.3	9.1	10.6	155	5.6	6.6	7.9	9.1	11.6
≥ 85	39	6.0	6.8	8.8	10.2	12.2	55	6.2	7.3	9.2	11.2	14.9
**2 min Step test (reps.)**												
60–64	223	77	88	96	106	116	254	83	96	106	115	124
65–69	237	69	84	98	110	118	282	65	81	96	108	116
70–74	207	68	82	93	106	115	226	64	80	94	106	116
75–79	203	64	74	89	101	114	190	59	70	85	99	110
80–84	154	48	65	78	90	102	142	49	60	78	93	104
≥ 85	34	62	64	77	86	104	43	40	54	65	77	96
**Body mass index (kg/m**^**2**^**)**												
60–64	227	21.9	23.2	25.1	26.7	28.5	271	21.0	22.4	24.2	26.8	28.5
65–69	242	21.0	22.8	24.8	27.1	28.9	300	20.7	22.2	24.7	27.0	29.3
70–74	225	21.2	23.1	25.0	27.5	29.3	244	20.2	22.2	24.5	27.3	29.8
75–79	214	20.5	22.2	24.8	27.0	29.5	220	19.8	21.7	24.3	26.6	29.6
80–84	164	20.4	21.9	24.2	26.2	27.6	161	19.6	21.1	23.5	27.1	29.2
≥ 85	42	20.1	22.2	24.3	26.2	29.3	61	18.4	21.1	24.0	26.2	30.6

## Discussion

The present study is the first to establish sex-specific FF norms in 5-year age intervals based on a pool of Chinese adults aged ≥ 60 years who lived in urban communities in mainland China. No harm or adverse effects occurred during test process. More than 90% of participants finished all the tests. These results indicate that the SFT is a feasible and safe test battery for independent Chinese older adults. A specific advantage of SFT is the design of the Step test as an aerobic endurance measurement alternative to the 6-min walking test. The Step test can be conducted without special requirements on the size of space. To increase test consistency and save human resources, the researchers highly recommend the incorporation of smart and wearable equipment in conducting all the tests.

The present results demonstrate an age-related degradation in each fitness dimension, which aligns with existing evidence^10–13^, and echoes the mainstream understanding of the interactive relationships between fitness and advancing age^16^. However, the decreasing rate differs between the considered fitness dimensions. Both upper and lower body flexibility in the present study exhibit the fastest degradation rates of all fitness components. This result aligns with those of many studies, such as those conducted in Hong Kong, Poland, and Portugal^10–12^. One important underlying cause could be related to the broader measurement range used in assessing flexibility. Since shoulder abduction and hip flexion ranges span more than 100°^20^, human body has considerable potential variations in flexibility.

No significant changes in BMI with increases in age were observed in this study. However, this does not mean no age-related changes in body compositions. Accumulated evidence has demonstrated the progressive reductions in muscle mass and height with advancing age. Even in people with stable weight, muscle is generally replaced with fat over time^21^. Given the non-sensitivity of BMI to the changes of muscle mass quality and fat location^21,22^, the suitability of BMI as a body composition indicator in the SFT warrants further study.

Regarding the turning point of declines in FF, no unified value for all fitness dimensions was observed. The present study indicated that the ages of 75 and 80 years could be two turning points in the FF for older adults by comparing the adjacent AG differences in each fitness test item. However, variations exist in sex-specific individual fitness components. Older women had significant degradations in dynamic balance and agility for each 5-year age interval from age 65 years onward, whereas similar changes were displayed in men from age 70 years onward. Both men (ES = 0.833) and women (ES = 0.720) had significant decreases in AC performance in AG2, indicating that age 65 years is the turning point for degradation in upper limb muscle strength in older adults. Based on the present findings, it is thus recommended to devise sex-specific exercise programmes targeting on different fitness dimensions so as to improve functional reserve capacity and independence in later years.

Sex differences were found in the participants’ overall fitness levels but not in each fitness test item. For example, with the exception of participants aged 65 to 69 years, nonsignificant differences in performance between the sexes were found in CS test for each AG. This aligns with the results from the aforementioned Hong Kong study^10^ but somewhat differs from the results of studies from Poland^11^, Portugal^12^, and the United States^13^, where men significantly outperformed women in the CS test. The non-significant sex differences in CS test of this study however, could be related with the cultural phenomenon that older women tend to be more active than age-matched older men in urban areas of China^10^. Just as reflected by the unique Chinese morning and evening exercises in public areas—a routine for countless older adults in mainland China, older women often comprise a greater proportion of the older adults who engage in this practice. Moreover, the most obvious sex differences were exhibited in the flexibility tests, wherein women performed better than men in every age group (BS test: ES = 0.291–0.693; SR test: ES = 0.393–0.729). Aside from these potential differences between men and women’s levels of physical activity, the higher performance levels of women in the flexibility test could be attributed to the differences between men and women’s tissue architecture and skeletal tissue morphologies^23^.

This study is the first to establish FF norms using the SFT battery in older adults of mainland China; this brings valuable information and will further improve the mainstream understanding of the fitness level of China’s ageing population. At the same time, the present results can be used for the international comparisons of FF with older adults from other countries or regions and with different cultural backgrounds. Participants of this study were 60 to 98 years old; fitness data with such a wide age range would be informative to assist policy-makers and professionals to promote successful aging for older adults, especially for those oldest old (≥ 85 years). In addition, results from this study can allow individual assessment of FF status against peers of the same sex and help early identification of losing independence.

This study has several limitations that may restrict the applicability of the results. First, all the participants were recruited from urban community senior centres, were apparently healthy, and regularly engaged in outdoor activities. Thus, the present normative data warrants further study when generalising to older adults who live in nursing homes, hospitals, or rural areas (e.g., China’s underdeveloped countryside). Secondly, data errors may persist even though standardised training workshops were conducted to ensure test accuracy and consistency between testers. The authors highly recommend the creation of test equipment for data recoding using smart technology. Thirdly, researchers applied convenience sampling method to recruit participants and implemented no limitations on participant enrolment from each senior centre. Therefore, sample size differences exist in the comparison groups. Lastly, although the cross-sectional study design is frequently used to study age-related changes in physical fitness, the changes with age may be underestimated when compared to the changes found with a longitudinal design.

In conclusion, this study established functional fitness norms using five percentiles for adults aged ≥ 60 years who lived in urban communities in mainland China. Progressive degradation in FF was observed with age, and ages of 75 years and 80 years were identified as two potential turning points in FF decline. Differences between the sexes varied considerably among AGs and fitness components. Future studies should be conducted to explore health-based FF criteria in older adults.
